# Supplementary material for: Identification of HSP90 inhibitors as a novel class of senolytics
Source: Nat Commun. 2017 Sep 4;8:422. doi: 10.1038/s41467-017-00314-z (PMC5583353; doi:10.1038/s41467-017-00314-z)
Supplement: Supplementary file 1 — Supplementary Information [file 41467_2017_314_MOESM1_ESM.pdf]

File Name: Supplementary Information
Description: Supplementary Figures and Supplementary Tables

File Name: Peer Review File
Description:

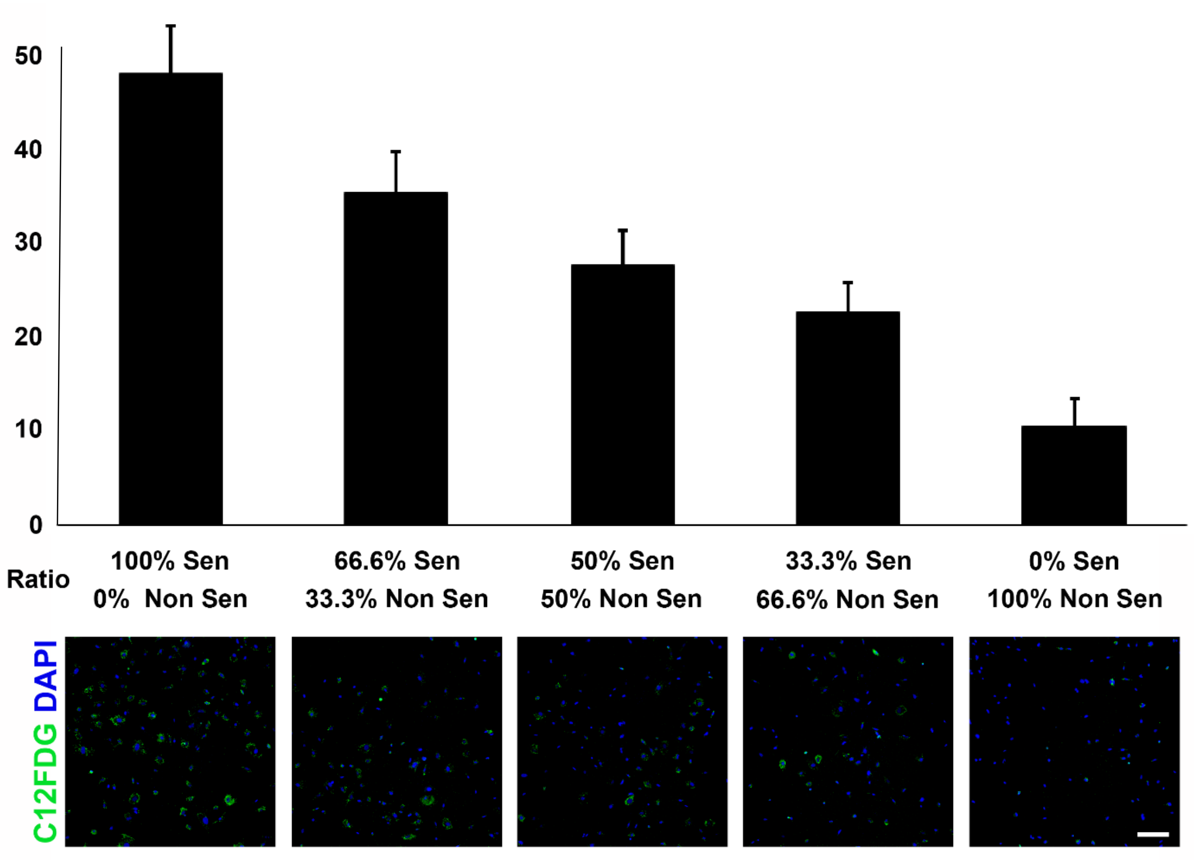

Supplementary Figure 1. Titration of senescent MEF cells. Different ratios of senescent and non-senescent MEF cells were plated together as indicated and analyzed with the IN Cell Analyzer 6000 24 hours later. Representative images of *Ercc1*$^{-/-}$ MEF cultures measuring senescence-associated β-gal (SA-ß-Gal) activity using C_{12}FDG and DAPI staining are shown below each graph. Error bars indicate SD for n=2, scale bar indicates 200μm.

Supplementary Table 1. Summary of senescence markers used in *Ercc1*$^{-/-}$ MEFs

Senescent Marker	SA-β–gal (% cell senescence)			Cell Size		Cell Cycle (x-fold)		SASP (positive)
Method	X-gal	Flow	Incell	Diameter (um)	Volume (pl)	p16	p21	IL-6^{+}
Non-senescent WT MEF cells	10%	6%	13%	20.2	4.3	1.1	n.d.*	n.d.*
Non-senescent *Ercc1*$^{-/-}$ MEF cells	22%	16%	15%	22.4	5.8	1.0	1.0	10%
Senescent *Ercc1*$^{-/-}$ MEF cells	51%	56%	48%	27.3	10.7	6.7	2.7	50%

*n.d.=not determined

Supplementary Table 2. Primary screen of autophagy regulators.

Rapamycin	Timosaponin A-III	NVP-BEZ235	PI-103	AS605240	LiCl	L690,330	Carpamazepine
Na-Valproate	Verapamil-HCl	Loperamide HCl	Amiodarone HCl	Nimodipine	Nitrendipine	Niguldipine	Penitrem A
Ionomycin	Rotenone	TTFA	Fluspirilene	Trifluoperazine·2 HCl	Sorafenib tosylate	Niclosamide	Rottlerin
Caffeine	Metformin·HCl	Clonidine·HCl	Rilmenidine	2',5'-Dideoxyadenosin	Suramin·6Na	Pimozide	STF-62247
Spermidine	FK-866	Tamoxifen citrate	Minoxidil	Imiquimod	Imatinib mesylate	AG112	SU11652
SB202190	Brefeldin A	Tunicamycin	Thapsigargin	A23187	Capsaicin	Dihydrocapsaicin	Glucosamine HCl
DTT	Deoxycholate·Na	ABC294640·HCl	Licochalcone A	Curcumin	Plumbagin	6-Gingerol	Akt Inhibitor X·HCl
PMSF	MG132	ALLN	7-Ketocholestero	17-AAG	Geldanamycin	C1	MZ36
Rockout	Go6850	2-Deoxyglucose	Etoposide	SMER28	Trehalose	C2-dihydroceramide	Temozolomide
Resveratrol	Staurosporine	Bafilomycin A1	3-Methyladenine	LY294002	Wortmannin	SP600125	Chloroquine
Hydroxychloroquine	Norclomipramine·HCl	(±)Bay K8644	Forskolin	Dibutyryl cAMP·Na	Rolipram	8-CPT-cAMP·Na	EHNA·HCl
SB-216763	Tolazamide	Quinine HCl·2H2O	AICAR	PD-98059	Anisomycin	Cycloheximide	Pifithrin-μ
Nocodazole							

Autophagy activator (senomorphic potential)
Autophagy activator (senolytic potential)
Autophagy inhibitors (senolytic potential)
Autophagy activator (cell toxicity)
Lysosomal inhibitors

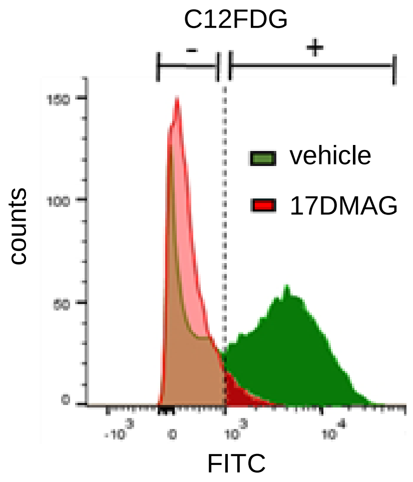

Supplementary Figure 2. Histogram of senescent *Ercc1*$^{-/-}$ cells treated with 100 nM 17DMAG or vehicle only. Green histogram indicate control cells, red histogram shows 17DMAG treated cells. Dark shades indicate $C_{12}FDG^{+}$ cells, light shades indicate $C_{12}FDG^{-}$ cells. Dotted line indicate cut-off between senescent $C_{12}FDG^{+}$ and non-senescent $C_{12}FDG^{-}$ cells.

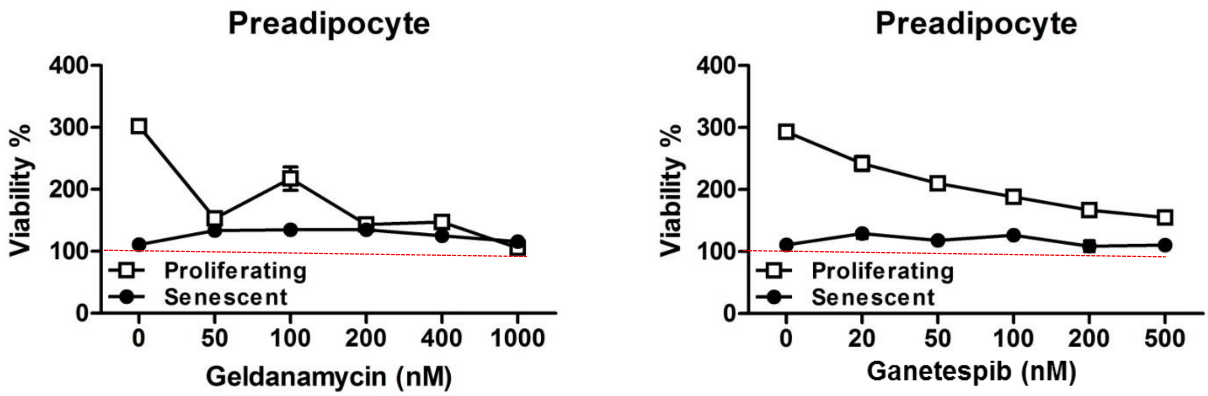

Supplementary Figure 3. Viability of preadipocytes treated with HSP90 inhibitors geldanamycin and ganetespib. Proliferating and senescent preadipocytes were exposed to different concentrations of geldanamycin (5-1000nM) and ganetespib (5-800nM). After 72 hrs, the number of viable cells were measured. The red line denotes plating densities on day 0 of non-dividing senescent (set to 100%) as well as proliferating non-senescent cells (also set to 100%). Plotted are the means of five replicates at each concentration. Senescence was induced by 10 Gy ionizing radiation.

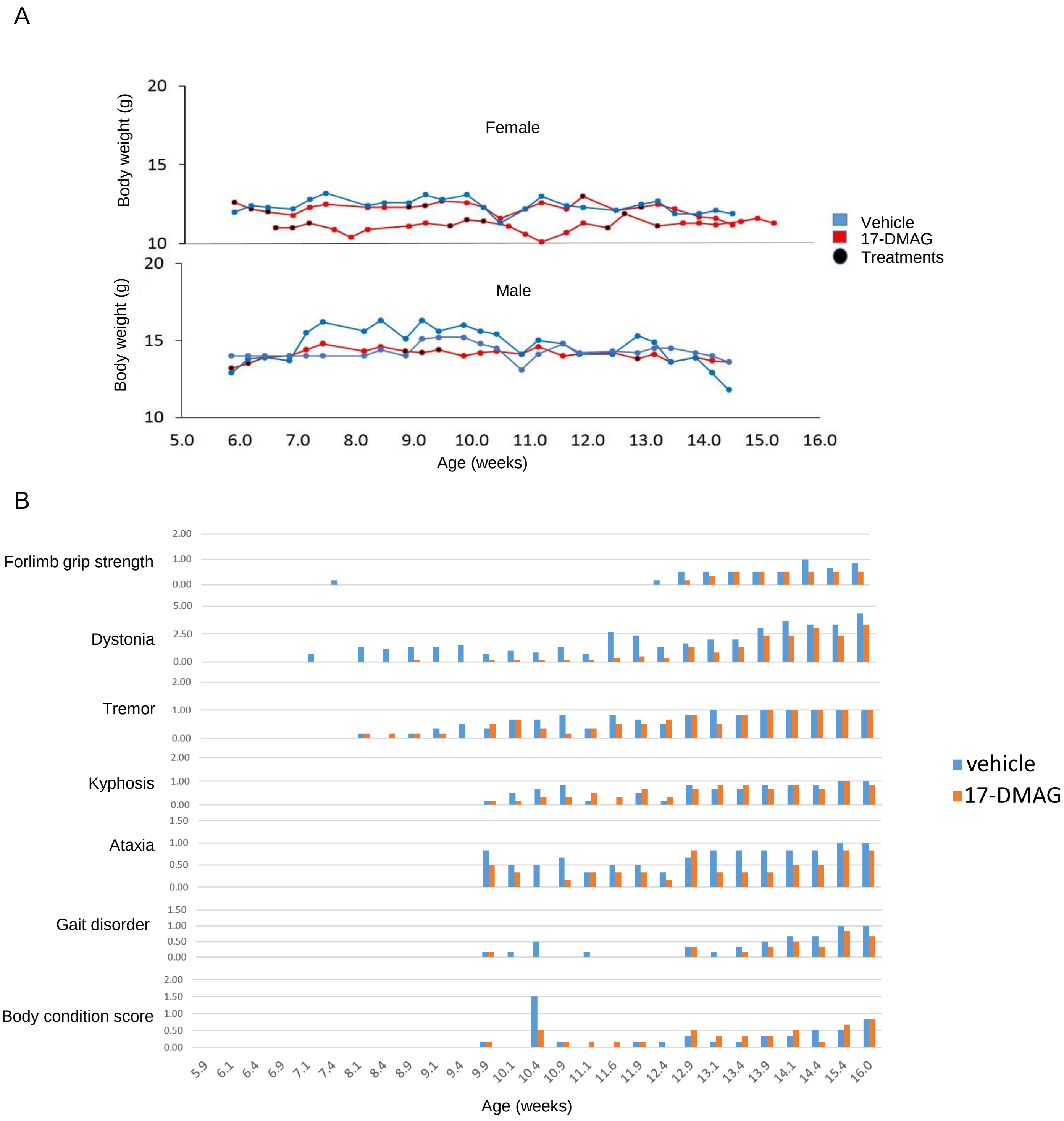

Supplementary Figure 4. **(A)** Weight Changes in 17-DMAG or vehicle treated mice. Body weight curves for male (lower graph) and female (upper graph) mice treated with 17-DMAG (red) or vehicle only (blue) are shown. Black dots indicate drug treatment. **(B)** Onset of age-related symptoms in 17-DMAG or vehicle treated *Ercc1*$^{-/\Delta}$ mice. The height of the bar indicates the severity of the symptom at a particular age. Many symptoms were delayed in onset in the *Ercc1*$^{-/\Delta}$ mice treated with 17-DMAG (blue) compared to siblings treated with vehicle only (orange) including dystonia, forelimb grip strength, gait disorder, ataxia and tremor.

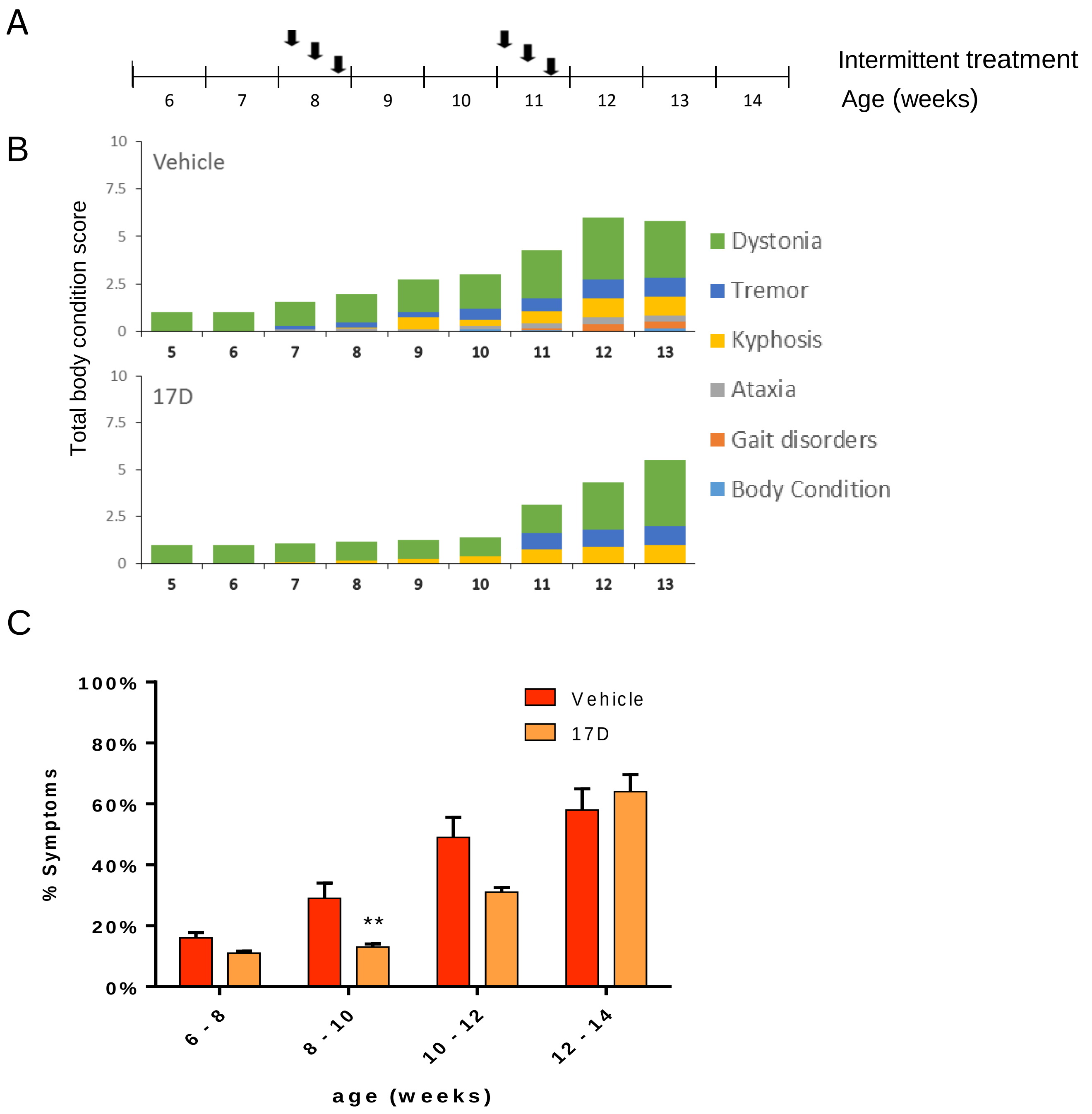

Supplementary Figure 5. (**A**) Schematic diagram of the short term *in vivo* treatment regiment. Animals were treated with 10 mg/kg 17-DMAG by oral gavage 3 times per week, every 3 weeks at 8 and 11 weeks of age. Five symptoms associated with frailty and aging were measured in the mice, once each week. (**B**) Graphed is the average age at onset of each symptom (appearance of a colored bar) and severity (height of the bar) for the group of $p16^{Luc/+};Ercc1^{-/\Delta}$ mice treated with vehicle or 17-DMAG. The composite height of the bar is an indication of the overall health (i.e., body condition score with a higher value being worse). (**C**) Comparison of age-related symptoms between cohorts of mice treated with the HSP90 inhibitor or vehicle only over time. The average fraction of total symptoms appearing in each age group is plotted. An increase in the percent of symptoms indicates a decrease in health. n=4 mice per treatment group (n=2 for female and n=2 for male), error bars indicate SEM, * $p<0.05$, ** $p<0.01$..

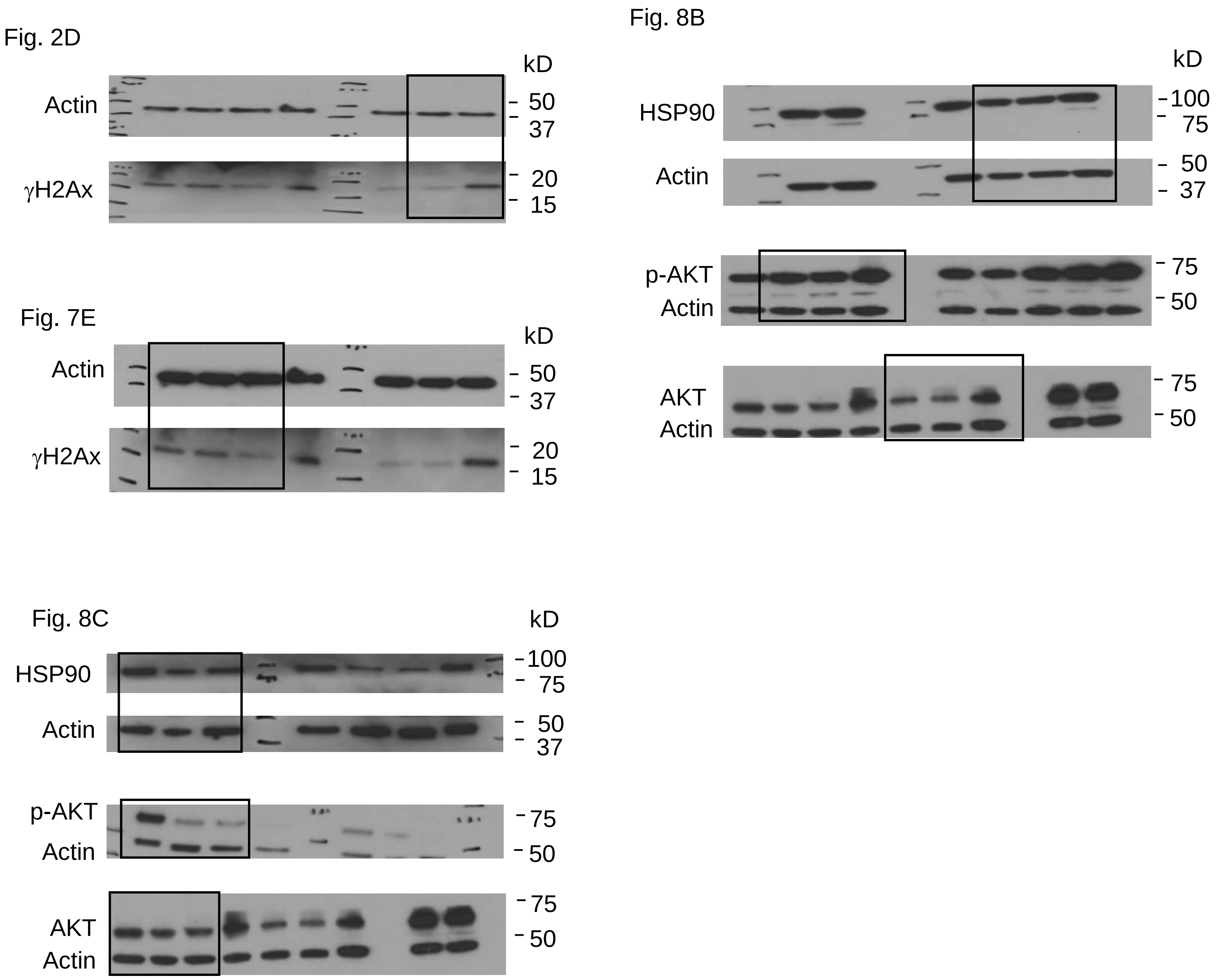

Supplementary Figure 7. Original Westernblots for Fig 2D, 8B and 8C.

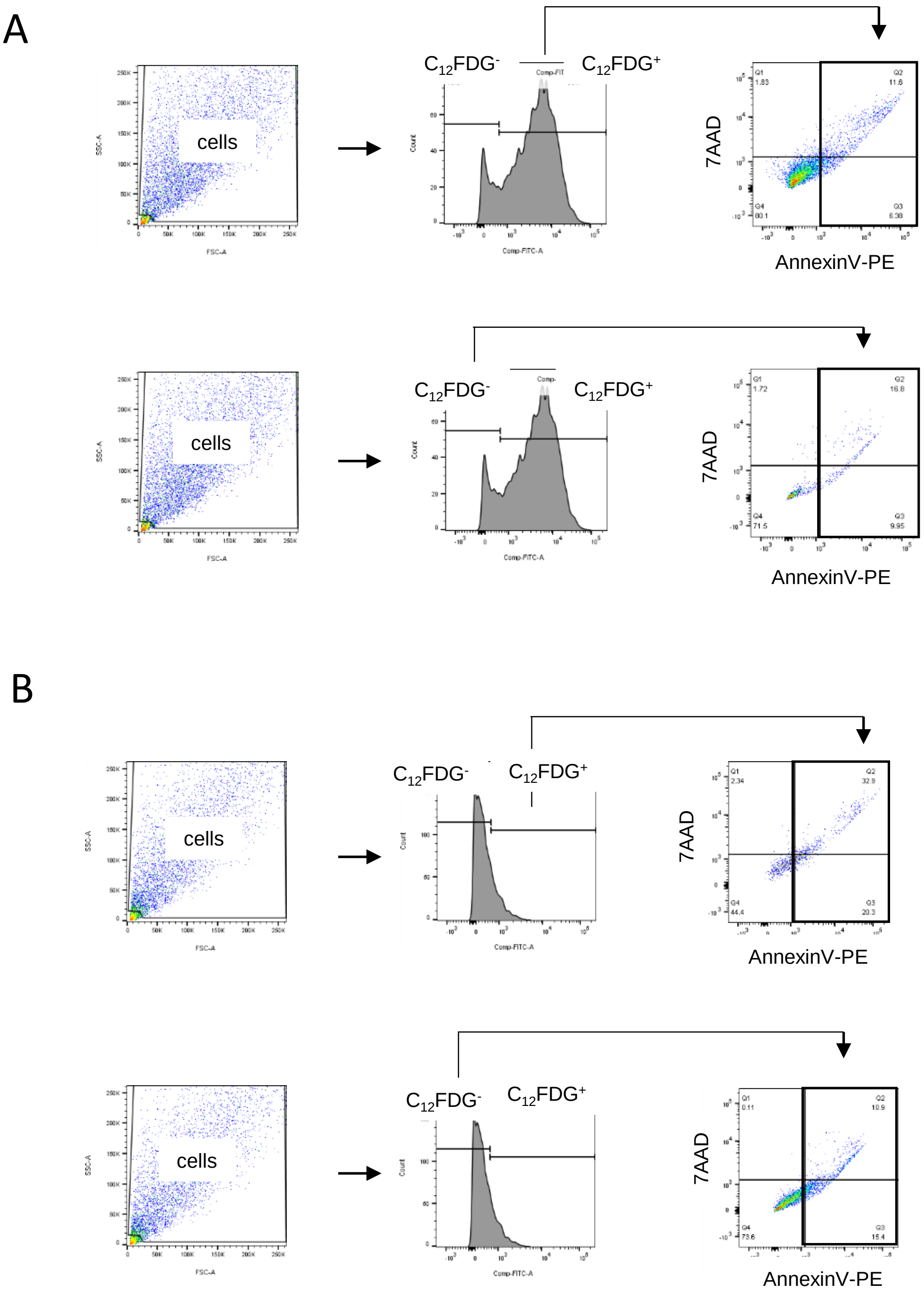

Supplementary Figure 8. Gating Strategy for C_{12}FDG-AnnexinV-7AAD Flow Cytometry. A two-parameter display of FSC *versus* SSC was set up to exclude subcellular debris. In a green fluorescence histogram C_{12}FDG fluorescence intensity was depicted in log scale. The percentage of positive cells was estimated by dividing the number of events within the bright fluorescence compartment by the total number of cells in the histogram. To estimate the number of live cells in SA-β-Gal positive and negative cells the subpopulation analyzed (C_{12}FDG positive cells or C_{12}FDG negative cells) was depicted on a two-parameter display of PE *vs*. PE-Cy5. The cells that were considered alive were those negative for PE (Annexin V-PE) and PE-Cy5 (7-AAD), AnnexinV-PE positive cells were considered apoptotic..(A) Untreated senescent MEF cells (B) 17DMAG treated cells.
